# Validity of Broselow tape for estimating the weight of children in pediatric emergency: A cross-sectional study

**DOI:** 10.3389/fped.2022.969016

**Published:** 2022-08-16

**Authors:** Shuzhen Zhu, Jihua Zhu, Hongqin Zhou, Xiuping Chen, Jianfeng Liang, Lijun Liu, Caidi Zhang, Yingying Zhao, Yanyan Chen, Xiao Wu, Sheng Ye, Kewen Jiang

**Affiliations:** ^1^Emergency Department, The Children's Hospital, Zhejiang University School of Medicine, Hangzhou, China; ^2^Department of Nursing, The Children's Hospital, Zhejiang University School of Medicine, Hangzhou, China; ^3^Statistics Office, The Children's Hospital, Zhejiang University School of Medicine, Hangzhou, China; ^4^Department of Child Psychology, The Children's Hospital, Zhejiang University School of Medicine/National Clinical Research Center for Children's Health and Diseases/National Regional Children's Medical Center, Hangzhou, China

**Keywords:** Broselow tape, pediatric emergency, cross-sectional study, weight estimation, China

## Abstract

**Objective:**

To assess the validity of the Broselow tape in estimating the weight of Chinese children in pediatric emergency.

**Methods:**

A cross-sectional study was conducted in the emergency department of the Children's Hospital of Zhejiang University School of Medicine (Hangzhou, Zhejiang Province, China) in March 2022. Broselow tape was used to estimate weight and its validity was compared with the advanced child life support (APLS) method.

**Results:**

The study included 442 children (mean age: 48 months; male-to-female ratio: 1.13:1). The < 10, 10–19 and > 19-kg groups included 44, 257, and 141 children, respectively. The color concordance rates of the Broselow tape-estimated weight in the three groups were 56.8, 57.2, and 68.1%, respectively. The percentage of weight estimations within 10% of actual weight were 65.8% (59.1, 65.8, and 68.1% for the <10, 10–19 and > 19-kg groups, respectively) and 44.8% (40.9, 50.6, and 35.5% for the < 10, 10–19 and > 19-kg groups, respectively) using the Broselow tape and the APLS method, respectively. The correlation between the Broselow tape estimated weight and actual weight was *r* = 0.931 (*P* < 0.0001, 95% CI: 0.918–0.943), while the correlation between actual weight and the APLS method calculated weight was *r* = 0.883 (*P* < 0.0001, 95% CI: 0.861–0.902). The mean percentage error using the Broselow tape was 1.0 ± 12.0% (*P* < 0.001 vs. −7.2 ± 17.2% of the APLS method).

**Conclusion:**

The Broselow tape may be an available method for predicting the weights of Chinese children in pediatric emergency.

## Introduction

Compared with adults, the dosage, infusion volume, equipment, etc. required for diagnosis and treatment of children are commonly calculated/evaluated based on weight. In pediatric emergency wards, there are often critical children with shock, severe trauma, respiratory arrest or circulatory failure who need immediate treatment or management, but it can be very difficult to obtain an accurate weight value, and an inaccurate weight estimation is likely to lead to wrong dosage, which will lead to serious consequences, and it is also one of the most common errors of improper medication in emergency wards. Hence, a rapid estimation of the children's weights is very essential to ensure the safety and accuracy of the treatments (1). Currently, the most frequently used methods for estimating the weight of pediatric emergency patients include parents or medical staff estimation, age-based formula, and length-based method (2). One-dimensional methods usually fail because they do not consider the variability in weight-for-age and weight-for-length and pediatric obesity (3). Two-dimensional methods can be more precise (4).

There are also precalculated estimation systems, such as the Broselow tape, advanced pediatric life support (APLS) method, devised weight estimation method (DWEM) method, Oakley table, Traub-Johnson method and Traub-Kichen method. Among them, the Broselow tape method has the longest history and the highest accuracy, and is the most widely used (4). The Broselow tape is a tape measure based on color-coding with length. The tape ruler is divided into nine colors and is suitable for children weighing 3 to 36 kg and measuring 47 to 143 cm. Moreover, the Broselow tape provides medical instructions, including drug dose, equipment model and voltage level during defibrillation. It is recognized as the standard of emergency treatment for children in most Western medical textbooks and publications and is recommended by advanced pediatric life support (APLS) (5, 6). Still, the most important issue with weight estimation methods is that these methods display insufficient accuracy and consistency among the different populations (7). The data that was used to design the Broselow tape was based on the National Center for Health Statistics (NCHS) and the national experimental survey on health and nutrition (NHANES) (4), which does not include data of Chinese children.

Therefore, this study aimed to evaluate the validity of the Broselow tape in estimating the weight of Chinese children.

## Methods

### Study design and participants

A cross-sectional was conducted in the emergency department of the Children's Hospital of Zhejiang University School of Medicine (Hangzhou, Zhejiang Province, China) in March 2022. The hospital admits children from Zhejiang province and surrounding provinces.

The inclusion criteria were children from newborn to 12 years old and voluntarily participating in the study. The exclusion criteria were A. children's height < 47 cm or > 143 cm and weight < 3 kg or > 36 kg; B. serious conditions preventing the actual weight from being measured, such as shock, severe trauma, respiratory arrest or circulatory failure; or C. specific conditions like amputation, cerebral palsy, microcephaly, dehydration, edema, growth hormone deficiency, severe joint contracture, or nerve defect that might seriously affect their weight and/or height. All subjects who met the inclusion and exclusion criteria were consecutively included. This study was approved by the ethics of the Children's Hospital of Zhejiang University School of Medicine (#2022-IRB-029). Written informed consent was obtained from the children > 8 years old or their legal guardians.

### Weight measurement

The actual weight of the children was measured using the RGZ-12-RT children's scale produced by Wuxi Weigher Factory Co., Ltd. The standard calibrated model 376 infants scale produced by SECA Medical Measurement System (Hangzhou) Co., Ltd. was used for infants < 2 years old. The subjects wore only light clothes and no shoes, and their weight was accurate to 0.1 kg.

The 2017 Edition A, Drs. Broselow^®^ Tape was used to measure the height; the tape has nine color zones: gray (3–5 kg), pink (6–7 kg), red (8–9 kg), purple (10–11 kg), yellow (12–14 kg), white (15–18 kg), blue (19–23 kg), orange (24–29 kg) and green (30–36 kg). In the supine position, the head was kept in a neutral position without pillows, the knees were kept straight, and the heels were 90° perpendicular to the bed. The length from the top of the head to the sole of the feet was measured. The difference between the color corresponding to the actual weight on the Broselow tape and the estimated weight was recorded.

The children's age and sex were recorded. The weight (kg) was also calculated according to the formula method of APLS (8) validated for Chinese children (9): 1–6-month-old weight = 3 + months × 0.7; 7–12-month-old weight = 6 + months × 0.25; 1–12-years-old weight = age × 2 + 8.

According to the actual measured weight, the children were divided into three groups: < 10-kg, 10–19-kg and > 19-kg, which corresponds to the color zones of the Broselow tape (gray, pink and red vs. purple, yellow and white vs. blue, orange and green), respectively. The same nurse measured the children's height and weight. Body mass index (BMI) was calculated: weight (kg)/[height (meter) × height (meter)].

### Sample size

The sample size was calculated using the Cochran formula, *n* = f (α, β) × 2s^2^/δ^2^. Based on the 95% confidence interval, which was equivalent to the confidence coefficient of 1.96, n was the minimum sample size, α was 0.05, β was 0.9, efficiency was 1-β = 0.1, δ was the minimum difference (10%), and s was the standard deviation (*s* = 20). Therefore, *n* = 10.5 × 2 (20) 2 / (10) 2 = 10.5 × 800/100 = 10.5 × 8 = 84. Therefore, the minimum sample size for each age group was 84, and the minimum sample size was 252.

### Statistical analysis

SPSS 23.0 (IBM, Armonk, NY, USA) and MedCalc 17.2 software (MedCalc Software bvba, Ostend, Belgium) were used for statistical analysis. All continuous data conformed to the normal distribution (according to the Kolmogorov-Smirnov test); they were expressed as means ± standard deviation (SD) and were analyzed using ANOVA. The categorical data were expressed as n (%) and analyzed using the chi-square test. Color zones consistency referred to whether the color corresponding to the actual weight on the Broselow tape was consistent with the color of the estimated weight measured by the Broselow tape. The deviation was the interval difference between the two colors. Accuracy was evaluated by percentage error; a small absolute percentage error indicated a high accuracy. Percentage error = (estimated weight - measured weight) / measured weight × 100%. The percentage of weight estimations within 10% (P10) and 20% (P20) of actual weight for the three weight groups was calculated. The difference between the color corresponding to the actual recorded weight on the Broselow tape and the color predicted by height was calculated. The Bland-Altman analysis was performed to assess the agreement between the two methods. Two-sided *P*-values < 0.05 were considered statistically significant.

## Results

Ultimately, 498 children aged 1 to 12 participated in this study. Fifty-six children were excluded because of height > 143 cm or weight > 36 kg, and 442 were included in the analysis. The ratio of males to females was 1.13:1. The three groups with a weight of < 10-kg, 10–19-kg and > 19-kg included 44, 257 and 141 children, respectively. The BMI of the three weight groups is 16.2 ± 1.5, 15.8 ± 2.0 and 16.4 ± 2.1 respectively (See Table 1).

**Table 1:** Characteristics of the children (*n* = 442).

**Characteristic**	**Actual measured weight grouping**			
	** <10-kg group** **(*n* = 44)**	**10–19-kg group** **(*n* = 257)**	**> 19-kg group** **(*n* = 141)**	**Total** **(*n* = 442)**
**Age (months)**	5.0 ± 9.7	36.0 ± 15.4	72.0 ± 19.3	48.0 ± 27.0
	(1.0–48.0)	(7.0–84.0)	(36.0–132.0)	(1.0–132.0)
**Sex**				
**Female**	22 (50.0)	113 (44.0)	73 (51.7)	208 (47.1)
**Male**	22 (50.0)	144 (56.0)	68 (48.3)	234 (52.9)
**Measured weight (kg)**	7.5 ± 1.7	15.5 ± 2.4	22.0 ± 4.2	17.0 ± 6.0
	(4.5–9.8)	(10.0–19.0)	(19.2–36.0)	(4.5–36.0)
**Measured height (cm)**	68.0 ± 5.9	101.0 ± 9.9	122.0 ± 9.0	104.0 ± 18.1
	(53.0–80.0)	(71.0–117.0)	(98.0–138.0)	(53.0–138.0)
**Broselow tape estimated weight (kg)**	8.0 ± 1.5	16.0 ± 2.8	24.0 ± 4.4	17.0 ± 6.0
	(4.0–11.0)	(9.0–22.0)	(15.0–34.0)	(4.0–34.0)
**Weight calculated by APLS method (kg)**	6.5 ± 2.5	14.0 ± 2.7	20.0 ± 3.2	16.0 ± 4.9
	(3.7–16.0)	(7.8–22.0)	(14.0–30.0)	(3.7–30.0)
**BMI**	16.2 ± 1.5	15.8 ± 2.0	16.4 ± 2.1	16.1 ± 2.0
	(14.5–20.1)	(10.7–23.7)	(11.8–29.2)	(10.7–29.2)
**IQR**	16.2	15.6	16.2	14.9
	(15.4, 17.1)	(14.7, 16.9)	(15.1, 17.3)	(15.9, 17.0)

Data are presented as mean ± standard deviation (min-max) or n (%). APLS, advanced pediatric life support. BMI, body mass index. IQR, interquartile range. BMI IQRs are presented as median (25%, 75%).

According to the actual weight, the consistency between the corresponding color on the Broselow tape and the color zone predicted by the height of the Broselow tape is shown in Table 2. The overall color zone consistency of the three groups was 60.6%, among which the color consistency of the gray and blue areas was ≥ 75%. Color mismatches of the Broselow tape was mainly one zone higher than the actual weight.

**Table 2:** Relationship between the estimated color zone of the Broselow tape and the color corresponding to the actual weight on the Broselow tape.

**Broselow tape color zone**	**Zone on the Broselow tape according to actual weight (*n*, %)**					
	**Same zone**	**Color error below one grid**	**Color error below two grids**	**Color error higher than one grid**	**Color error higher than two grids**	**Total (*n*)**
< **10-kg group**	25 (56.8)	3 (6.8)	0	16 (36.4)	0	44
**Gray**	3 (75.0)	0	0	1 (25.0)	0	4
**Pink**	12 (60.0)	0	0	8 (40.0)	0	20
**Red**	10 (50.0)	3 (15.0)	0	7 (35.0)	0	20
**10–19-kg group**	147 (57.2)	40 (15.6)	1 (0.4)	65 (25.3)	4 (1.6)	257
**Purple**	9 (39.1)	4 (17.4)	0	9 (39.1)	1 (4.4)	23
**Yellow**	30 (50.0)	7 (11.7)	1 (1.7)	19 (31.7)	3 (5.0)	60
**White**	108 (62.1)	29 (16.7)	0	37 (21.3)	0	174
> **19-kg group**	96 (68.1)	19 (13.5)	2 (1.4)	23 (16.3)	1 (0.7)	141
**Blue**	53 (75.7)	9 (12.9)	0	7 (10.0)	1 (1.4)	70
**Orange**	32 (57.2)	6 (10.7)	2 (3.6)	16 (28.6)	0	56
**Green**	11 (73.3)	4 (26.7)	0	0	0	15
**Total**	268 (60.6)	62 (14.1)	3 (0.7)	104 (23.5)	5 (1.1)	442 (100)

**Figure 1:**
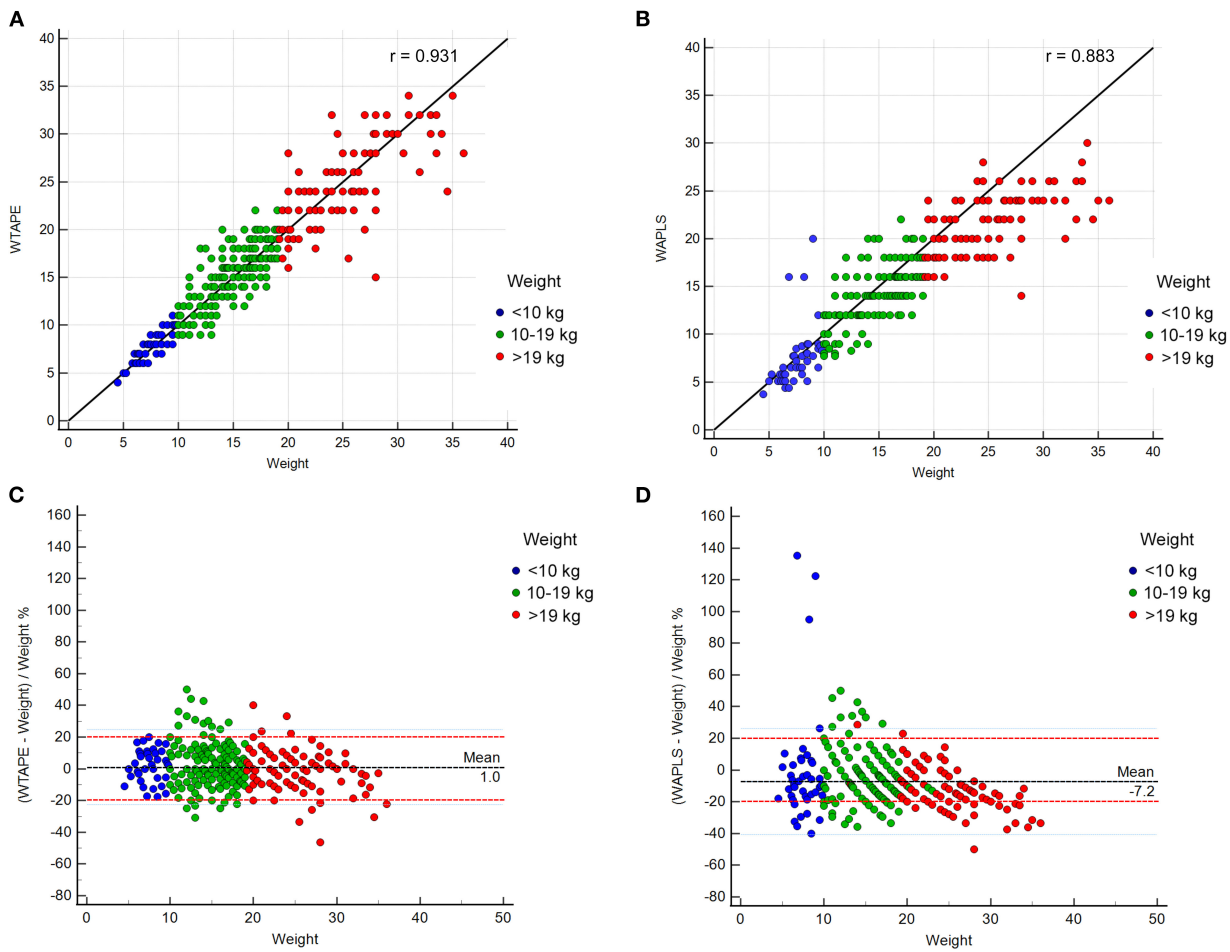
**(A)** Correlation analysis between the Broselow tape estimated weight and actual weight; **(B)** Correlation analysis between the advanced pediatric life support (APLS) method-calculated weight and actual weight. **(C)** Percentage error between the Broselow tape estimated weight and actual weight; **(D)** Percentage error between the formula estimated weight and the actual weight. WTAPE, Broselow tape estimated weight; WAPLS, advanced pediatric life support method-calculated weight.

The relationships between the weight estimated using the Broselow tape and the APLS method and actual weight are shown in Figures 1A,B. The correlation between the Broselow tape estimated weight and actual weight was *r* = 0.931 (*P* < 0.0001, 95% CI: 0.918–0.943). The correlation between actual weight and the APLS method calculated weight was *r* = 0.883 (*P* < 0.0001, 95% CI: 0.861–0.902).

In all children, the P10 were 65.8% (92.8% for P20) and 44.8% (83.7% for P20) using the Broselow tape and the APLS method, respectively (Table 3, Figures 1C,D). The proportions of children with P10 using the Broselow tape were the lowest in the < 10-kg group (59.1%) and the highest in the > 19-kg group (68.1%); while the proportions of children with P20 was lowest in the 10–19-kg group (91.8%) and highest in the < 10-kg group (100%) group (Table 3). Furthermore, the mean percentage error (MPE) using the Broselow tape was close to zero (1.0 ± 12.0%), and its levels in all children and two weight groups (10–19-kg and > 19-kg) were significantly different from that using APLS method (−7.2 ± 17.2% in all children, −5.1 ± 14.1% of 10–19-kg group and −13.0 ± 11.3% of > 19-kg group; *P* < 0.001, respectively) (Table 3).

**Table 3:** Percentage error of the Broselow tape and formula estimated weight.

	**Broselow tape**			**APLS**		
	**MPE**	**P10**	**P20**	**MPE**	**P10**	**P20**
**Weight**						
< **10-kg**	1.5 ± 10.3	26 (59.1)	44 (100)	−1.0 ± 35.5	18 (40.9)^**^	36 (81.8)^**^
**10–19-kg**	1.8 ± 12.2	169 (65.8)	236 (91.8)	−5.1 ± 14.1^**^	130 (50.6)^**^	222 (86.4)^**^
> **19-kg**	−0.6 ±12.0	96 (68.1)	130 (92.2)	−13.0 ± 11.3^**^	50 (35.5)^**^	112 (79.4)^**^
**All**	1.0 ±12.0	291 (65.8)	410 (92.8)	−7.2 ± 17.2^**^	198 (44.8)^**^	370 (83.7)^**^

Data are presented as mean ± standard deviation or n (%); APLS, advanced pediatric life support. MPE, mean percentage error; P10, percentage of weight estimations within 10% of actual weight; P20, percentage of weight estimations within 20% of actual weight;

** P < 0.001 (compared to Broselow tape method).

## Discussion

The results showed a good linear positive correlation between the Broselow tape-estimated weight and the actual weight, and the Broselow tape method has better estimation accuracy than APLS method commonly used in China. Its MPE is close to zero and the P10/20 is high. Overall, the Broselow tape should be an available method for predicting the weights of Chinese children in pediatric emergency.

Physicians worldwide made various attempts to design weight estimation tools for children. There are many tools and formulas that can help medical personnel estimate weight. Currently, the method used more frequently in China is the APLS method (8) which had been validated for the Chinese children (9). Although the APLS method is very simple, fast and convenient, these estimations are crude and often inaccurate due to the differences in growth spurts, nutritional status and rates of obesity. Moreover, these formulas will no longer work when the children's age is unknown (10, 11).

In 1986, the World Health Organization (WHO) (12) proposed the DWEM method, which is based on a table according to length and body shape (thin, medium and fat). The accuracy was acceptable, but the body shape is subjective and variable among populations, introducing bias.

In 1988, Dr. Oakley drew up a table according to age and length, the estimated weight can be found on the table, which was once widely used in the emergency wards (13), but it was gradually replaced by the Broselow tape owing to its portability and ease of operation. Compared with age, body shape and other factors, height correlates best with weight (14). The Broselow tape and color partition system were analyzed using the data of the National Center for Health Statistics of the United States in the 1970s (5, 15). Nevertheless, the Broselow tape was designed using data from the United States of America. Other studies also examined the appropriateness of the Broselow tape in different populations, suggesting that it is a useful tool for estimating children's weight. A study of 2,358 American participants aged up to 16 years from a trauma registry (2002–2006) showed that the Broselow tape was an ineffective tool for predicting weight in more than 50% of pediatric participants (16), supported by Waseem et al. (17). Also in the United States of America, Abdel-Rahman et al. (18) showed that the proportion of children with P10 and P20 was 59% and 91%. In South Africa, Geduld et al. (19) showed that the mean difference between the actual weight and that predicted by Broselow tape was 0.9%, while Wells et al. (20) showed that the MPE was −3.8% between Broselow tape and the actual weight. In Kenya, the overall MPE for the actual weight and Broselow tape was −2.2% (21). In Nigeria, weight estimates obtained using the Broselow tape correlated better in children that are 6 years or younger compared to those in the older age categories (22). In Mexico, The Broselow tape-estimated weight was different from the scale weight by more than 10% in a substantial percentage of Mexican children; nevertheless, the MPE was < 3%, and Broselow tape color zone estimation was accurate in most subjects, and the P10 was 63.6% (23). Closer to China, Shah et al. (24) showed that the Broselow tape overestimated weight by > 10% in most Indian children; the weight overestimation was greater in children of the > 18 and 10–18 kg weight groups. In Nepal, the accuracy of the Broselow tape in estimating the weight of a child, accuracy decreases as the weight of the child increases (1). Still, data were lacking for the Chinese population.

The present study investigated the validity of the Broselow tape in the weight estimation of Chinese children in the emergency department of an upper-tertiary pediatric specialty hospital in Hangzhou, Zhejiang province. The results showed a good linear positive correlation between the Broselow tape-estimated weight and the actual weight. Moreover, the MPE of the Broselow tape method was 1%; and the proportions of children with P10 and P20 was significantly higher than the APLS method. Our results indicated that the consistency of the Broselow tape was better than the APLS, as supported by the literature from other countries (1, 22, 24). Is the result of the present study due to the inclusion of a uniform body shape population? Our results showed that the means, SD, skewness and kurtosis of BMI of all ages groups (male/female) are close to the data of Chinese population samples reported in the literature (25, 26). This shows that our study population is representative. In addition, the BMI of 26 children (5.9%) exceeded the percentile range of 3^rd^-97^th^ of the Chinese population (25, 26). Therefore, this study does not include a uniform body shape population. The proportions of children with P10 using the Broselow tape was the highest in the > 19-kg group; while the proportions of children with P20 was highest in the < 10-kg group, which differed from studies in other countries (1, 22, 24). The present results did not meet the acceptable accuracy of estimation (P20 > 95% and P10 > 70%), as has previously been suggested (4, 27). Different races, different economic levels and different geographical characteristics of different countries will lead to these differences. Indeed, Pukar et al. (1) (Nepalese children) and Iloh et al. (22) (Nigerian children) showed that the accuracy was better in the low weight zones. In Indian children, Shah et al. (24) showed that the accuracy was the lowest in the 10–18-kg group. In the present study, the Broselow tape overestimated the weight as the weight level was higher than the actual measured weight in all three weight groups (Table 1), supported by previous studies (24, 28, 29). Basing on the data of P20 using the Broselow tape, we found that the total overestimated ratios (4.5%, 20/442) was higher than that of the underestimated ratios (2.7%, 12/442) in all children; for three weight groups, the underestimated ratios were the lowest in the < 10-kg group (0.0%) and the highest in the 10–19-kg and > 19-kg group (1.4%, respectively); while the overestimated ratios were the lowest in the < 10-kg group (0.0%) and the highest in the 10–19-kg group (3.4%) (22, 24, 30). Still, the exact reasons regarding why the consistency was better in the > 19-kg group are mostly unknown. We analyzed the BMI of these under/overestimated children using the Broselow tape method, and found that if the BMI < 3^rd^ or > 97^th^ of the percentile range, their weight would be underestimated or overestimated, respectively. It is urgent to do large samples study and to setup a manual correction method for these children (BMI < 3^rd^ or > 97^th^). It could be also related to the development of the children in the socio-economic and medical context of China. Although both the overestimated and underestimated ratios were 0.0% in the <10-kg group, its validity might have been compromised for the small sample size of this group.

This study had some limitations. Although the study center is a large tertiary center admitting patients from Zhejiang province and surrounding provinces, the study population might not represent all Chinese children, though the BMI of these population are in line with the current Chinese BMI data. The economic and social development level of Zhejiang province is advanced in China, and the residents' physique is similar to industrialized countries. The education level is high, and more attention is paid to the management of children's growth and development. In this study, 5.9% of children with BMI exceed the 3^rd^-97^th^ percentile range of the Chinese population, slightly <6%, which might lead to a higher estimation accuracy by the Broselow tape. In addition, because it was an observational study, sampling and measurement errors are possible. Furthermore, the wide range of exclusion criteria related to specific situations will results in the effectiveness of these children in pediatric emergency rooms has yet to be evaluated. Finally, the sample size is small of the < 10-kg group, its dada validity might have been compromised.

In conclusion, there was a positive correlation between the Broselow tape-estimated weight and the actual weight, and the Broselow tape-estimated method had a better estimation accuracy than that of the APLS method. The Broselow tape should be an available method for predicting the weights of Chinese children in pediatric emergency, despite its introduction will increase the additional cost of purchasing tape, and employees need to be trained to ensure the proper use of tape.

### Prior presentation

The results from this study were presented at Zhejiang Medical Association Pediatrics Branch academic annual meeting and new progress in diagnosis and treatment of pediatric diseases national continuing education class 2015, in hangzhou, china.

## Data availability statement

The original contributions presented in the study are included in the article/supplementary material, further inquiries can be directed to the corresponding authors.

## Author contributions

SZ, JZ, HZ, XC, SY, and KJ conceptualized and designed the study. SZ, LL CZ, YZ, YC, and XW coordinated and supervised the data collection. SZ and JL performed the statistical analyses. SZ drafted the initial manuscript, and takes responsibility for the paper as a whole. JZ, HZ, XC, SY, and KJ contributed substantially to its revision. All authors contributed to the article and approved the submitted version.

## Funding

The Natural Science Foundation of Zhejiang Provincial Department of Education Project (Y202045544), and the National Natural Science Foundation of China (81871012 and 81571263).

## Conflict of interest

The authors declare that the research was conducted in the absence of any commercial or financial relationships that could be construed as a potential conflict of interest.

## Publisher's note

All claims expressed in this article are solely those of the authors and do not necessarily represent those of their affiliated organizations, or those of the publisher, the editors and the reviewers. Any product that may be evaluated in this article, or claim that may be made by its manufacturer, is not guaranteed or endorsed by the publisher.
